# Friendly fire: Longitudinal effects of exposure to violent video games on aggressive behavior in adolescent friendship dyads

**DOI:** 10.1002/ab.21748

**Published:** 2018-01-24

**Authors:** Geert P. Verheijen, William J. Burk, Sabine E. M. J. Stoltz, Yvonne H. M. van den Berg, Antonius H. N. Cillessen

**Affiliations:** ^1^ Behavioural Science Institute Radboud University Nijmegen The Netherlands

**Keywords:** adolescence, aggressive behavior, peer influences, video games

## Abstract

Research on gaming effects has focused on adolescence, a developmental period in which peer relationships become increasingly salient. However, the impact of peers on the effects of violent gaming on adolescents has been understudied. This study examined whether adolescents’ exposure to violent video games predicted their own and their friend's aggression one year later. Among 705 gaming adolescents, 141 dyads were identified based on reciprocated best friend nominations (73.8% male, *M_age_* = 13.98). Actor‐Partner Interdependence Models indicated that adolescent males’ (but not females’) exposure to violent games positively predicted the aggression of their best friend 1 year later. This effect appeared regardless of whether the friends played video games together or not. The study illustrates the importance of peers in the association between violent gaming and aggression.

## 1 INTRODUCTION

Video games have become one of youths’ most popular pastimes. Along with their popularity, public concern has grown about the developmental risks of video games. The association between violence in video games and aggression especially has received much attention. In a recent statement, the American Psychological Association confirmed a link between playing violent games and aggression, but also called for more nuanced research on the characteristics of video games (APA, 2015). The current paper contributes to this call by investigating the *social context* in which violent video games are played.

### 1.1 Violent gaming and aggression

Several meta‐analyses have reported positive associations between exposure to violent games and aggression (Anderson et al., 2010; Greitemeyer & Mugge, 2014; but see Ferguson, 2015). This association is commonly explained through the General Aggression Model (GAM; Anderson & Bushman, 2002), which posits that aggressive behavior is based on idiosyncratic personal variables, such as gender or trait aggression, and situational or environmental variables, such as the amount of violence in a video game. Together, personal and situational variables increase individuals’ aggressive affect, aggressive cognitions, and physiological arousal. In turn, this impacts on appraisal and decision‐making processes (Bushman, 2016), leading to aggressive behavior in the short‐term. Furthermore, repeated exposure to violent media leads to changes in the chronic accessibility of aggression‐related knowledge structures, causing long‐term changes in individuals’ personality.

The GAM and other social‐cognitive models, such as Social Learning Theory (Bandura, 1973) and the Differential Susceptibility to Media Effects Model (Valkenburg & Peter, 2013), specify the moderating role of the social context for persistent and long‐term changes in aggression. A person's environment can encourage or discourage the use of aggression, which exacerbates or dampens the long‐term effects of exposure to media violence (Anderson et al., 2010). When exposure to violence increases a person's short‐term aggression, any positive responses by the environment to this newly adopted aggressive behavior might solidify the effect. These social influences can occur deliberately through explicit regulation of media use, or more candidly through the prevailing norms of family or peers (Valkenburg & Peter, 2013). Furthermore, not only does acceptance of aggressive norms increase the likelihood of lasting violent gaming effects, but also habitual exposure to violent games can increase normative acceptance of aggression (Krahé & Möller, 2004). Thus, the social environment plays an important role in enhancing or mitigating the effects of violent games on aggressive behavior (Gentile, 2011). This is especially true in adolescence, when peers are increasingly influential (Brown & Larson, 2009).

Adolescents are one of the biggest target demographics of the video game industry. At the same time, they may be particularly vulnerable to the effects of violent media (Kirsh, 2003). Maturation of brain regions in puberty leads to relatively higher reward‐seeking and lower impulse control (Steinberg, 2010). This may cause attraction to fast‐paced violent games and increased aggressive behavior afterwards due to difficulty in arousal‐regulation (Willoughby, Adachi, & Good, 2012). Adolescence is also accompanied by increased independence and reduced parental control of media use, which can increase exposure to violent media and games in particular as these are appealing due to a “forbidden fruit effect” (Bijvank, Konijn, Bushman, & Roelofsma, 2009). Thus, biological and psychological changes, accompanied by increased exposure to violent content, could enhance the impact of violent game effects in adolescence.

### 1.2 Social context of gaming

In contrast with the stereotypical image of the “lone gamer,” most teen gamers play with others. Video games seem to play a role in the development and maintenance of friendships, especially for males, as 42% plays games with friends on a weekly basis and 34% made friends while playing games online (Lenhart, Smith, Anderson, Duggan, & Perrin, 2015). Furthermore, a large body of literature demonstrates the importance of peers in shaping adolescents’ behavior (Brechwald & Prinstein, 2011). It is therefore surprising that the role of friends in the association between violent games and aggression has not yet been studied.

The *socialization effect* states that peers influence each other's behavior so that they become more similar over time. Indeed, affiliation with an antisocial peer increases adolescents’ own aggressive attitudes and behavior (Cohen & Prinstein, 2006). The socialization of aggression among adolescents may take place through *deviancy training*, a process in which peers reinforce each other's antisocial attitudes and behavior through positive affective behavior, such as laughing (Dishion, Eddy, Haas, Li, & Spracklen, 1997). This process may also occur when adolescents play violent video games. Friends reinforce behavior occurring on screen as they laugh, give advice, boast and encourage in‐game behavior (Stevens, Satwicz, & McCarthy, 2008). Social reinforcement through positive affect of violent behavior in a game could increase aggressive behavior in the future, both inside and outside of the gaming setting. Deviancy training may even occur from discussing a violent game with peers, regardless of whether adolescents played the game themselves.

### 1.3 Current study

The current study investigated the association between exposure to violent video games and aggression over a 12‐month period. Violent gaming of adolescents and their best friends was examined. We applied dyadic data analytic techniques to simultaneously estimate the effects of violent video games on changes in own aggressive behavior (actor effects) and friends’ aggressive behavior (partner effects), while adjusting for the similarity between friends’ aggressive behavior (Kenny, Kashy, & Cook, 2006). Analyses were performed separately for males and females, as recommended by the American Psychological Association Task Force on Violent Media (2015).

Three hypotheses were tested. First, based on the GAM (Anderson & Bushman, 2002), we expected that adolescents’ exposure to violent video games would predict an increase in their own aggressive behavior 12 months later (*actor effect)*. Second, based on deviancy training, we expected that exposure to violent video games would also predict the aggressive behavior of their best friend (*partner effect*). Third, we expected that the partner effect would be moderated by whether or not friends played video games together. We predicted that the partner effect would be stronger when friends play games together at least occasionally. If two friends only play games apart from one another, they are less likely to discuss or socially reinforce the violent in‐game behavior and deviancy training is expected to be minimal.

## 2 METHOD

### 2.1 Participants

The current study was part of the Kandinsky Longitudinal Study (KLS), a research project aimed at detecting youth who are at risk for social and emotional problems in secondary education (Stoltz, Cillessen, van den Berg, & Gommans, 2016). The KLS includes adolescents in 7th to 10th grade from one high school in the Netherlands. The current study used two waves of the KLS from November 2014 (T1) and November 2015 (T2). At T1, 1086 adolescents were recruited across 41 classrooms. Absenteeism, lack of consent, and unfinished questionnaires due to time constraints brought the total number of participants to 1,016 adolescents (51.2% female, *M_age_* = 14.16, SD = 1.27). Adolescents who indicated that they never play video games were removed from the sample (*n* = 311), bringing the final sample to 705 adolescents (33.5% female, *M_age_* = 14.07, SD = 1.29).

### 2.2 Procedure

Participants completed a computerized survey on aggression and gaming behavior, as well as measures of adjustment beyond the scope of the current study, in a 45–60 min assessment session in their classroom at school. Instructions were provided verbally and on the laptop. Adolescents sat at a private desk and talking was prohibited. There were always at least two researchers present during data collection.

The KLS project was formally requested by the school in 2010 in order to gain insight in students’ social and emotional problems. The school declared responsibility for the parental consent procedure before each wave of data collection. A letter describing the research procedure was sent to parents at the beginning of each school year. Parents could exclude their child from participation by responding to the letter. Thus, informed parental consent was obtained through the school. Adolescents provided assent each year at the start of the survey as well. This procedure was approved by the Institutional Review Board of the Behavioural Science Institute at Radboud University.

### 2.3 Measures

#### 2.3.1 Selection of best friend dyads

At T1, adolescents were presented with a roster of names from classmates and asked to nominate their friends, starting with their first best friend, second best friend, and so on. Two peers who reciprocally nominated each other as first best friends were identified as a best friend dyad. Out of 705 participants, 143 best friend dyads were identified at T1. Most dyads were same‐sex (104 males and 37 female). Two mixed‐sex dyads were omitted from further analyses, bringing the final number of best friend dyads to 141 (73.8% male, *M_age_* = 13.98).

#### 2.3.2 Gaming together or apart

In order to examine whether the partner effect of violent gaming on aggression would be stronger when friends actually played video games together, best friend dyads were assigned to one of two groups. Participants indicated how often they played video games together with the person they nominated as their first best friend, ranging from 0 (*never*) to 8 (*7 days a week*). When both members of a dyad answered this question with at least 1 (*less than 1 day per week*), they were considered a *gaming‐together* dyad. If at least one dyad member indicated 0 (*never*), the dyad was considered a *gaming‐apart* dyad^1^. For males, there were 66 gaming‐together dyads and 38 gaming‐apart dyads (63.5% and 35.5% of all gaming dyads respectively). Female best friend dyads were less evenly distributed with only 8 gaming‐together dyads and 29 gaming‐apart dyads (21.6% and 78.4% of all gaming dyads, respectively).

#### 2.3.3 Aggressive behavior

Aggressive behavior was measured at T1 and T2 with peer nominations of physical aggression, which is considered the most prevalent form of aggression in video games and commonly used in violent media research (Smith, Lachlan, & Tamborini, 2003). Adolescents received a roster with the names of their classmates and were asked to nominate “which person in your class kicks, pushes, or hits others?” They could name as many peers as they saw fit, but not themselves. The number of nominations received was counted for each participant and standardized within classrooms. A reciprocal transformation was applied to the aggression scores because they were positively skewed at T1 (skew = 1.91, *SE* = 0.15) and T2 (skew = 2.27, *SE* = 0.17).

#### 2.3.4 Exposure to violent games

Exposure to violent games was computed at T1 by multiplying participants’ average time spent playing video games per week with the average level of violence in the genres they played. Participants first indicated the number of hours spent gaming per week with two questions “During the week, how many hours do you play video games?” and “During the weekend, how many hours do you play video games?” Answers ranged from 0 (*I never play video games*) to 5 (*More than 4 hours per day*). The average time playing video games per week was estimated by multiplying the weekday score by five and adding the weekend day score multiplied by two.

Participants then chose all video game genres they played in the past 12 months from a list of 16 genres: *Action, First‐Person Shooters, Fighting, Survival Horror, Adventure, Sport, Simulation, Platformer, Racing, Rhythm, Puzzle, Massive Multiplayer Online games, Role‐Playing Games, Virtual World*, and *Social Network Games*. Up to three commercial titles were given as examples for each genre. Next, every genre received a score for the typical level of violence in it based on expert ratings (Busching et al., 2015). Four student raters with hands‐on gaming experience with each genre indicated how much violence each genre typically contains, ranging from 1 (*free of violent content*) to 5 (*high level of violent content*). Violent media depictions were defined as harming of humans, humanoid characters, or other beings by one or more media characters/players, typically presented through battle scenes or fights where characters hit, shoot at, injure, and/or kill others, where there is plenty of blood, and where scenes of injuring and killing others are presented in a realistic way (Busching et al., 2015). The mean score across raters was used as the violent content score of each genre. The average intraclass correlation coefficient (ICC) was 0.98, indicating excellent rater reliability, *F*(15, 45) = 48.28, *p* < .001. To compute the final score of exposure to violent games for each participant, the violent content score of all genres played was averaged and multiplied by the time spent playing video games per week. Standardized scores were used in all analyses.

## 3 RESULTS

### 3.1 Preliminary analyses

All preliminary analyses were performed using the “psych” package in Revelle (2016). Table 1 presents the means and standard deviations of all study variables separately for males and females. Welch's *t*‐tests revealed that males scored significantly higher on exposure to violent games than females, *t*(138.82) = 9.32, *p* < .001, *d* = 1.30. Males also showed more aggression than females at T1, *t*(241.31) = 10.05, *p* < .001, *d* = 1.42), and T2, *t*(173.36) = 7.31, *p* < .001, *d* = 1.10).

**Table 1:** Spearman's ρ, means, and standard deviations of main study variables for male and female dyads

	Males (*n* = 208)			Females (*n* = 74)		
Variable	1	2	3	1	2	3
1. Exposure to violent games	.20*	.06	.14	−.02	−.12	.17
2. Aggression T1	.01	.52***	.54***	−.09	.78***	−.02
3. Aggression T2	.17*	.31***	.38**	.05	−.02	.52**
*M*	60.72	.32	.24	25.20	−.43	−.41
*SD*	31.03	.06	1.02	26.00	.39	.26

Intrapartner correlations are above the diagonal, cross‐partner correlations are below the diagonal, and intraclass correlations are on the diagonal.

^*^ *p* < .05, **p < .01, ***p < .001.

Dyadic correlations between all study variables are also presented in Table 1 separately for males and females. Statistical significance of all correlations was adjusted based on the formulas provided by Griffin and Gonzalez (1995) to account for the indistinguishable nature of the friendship dyads. The intra‐individual (actor) correlations, which describe associations between main study variables of a single dyad member, are shown above the diagonals. Exposure to violent video games was not associated with males’ or females’ own aggression at T1 or T2. There was a positive correlation between aggression at T1 and aggression at T2 for males, but not females.

Correlations describing inter‐individual (partner) associations are presented below the diagonals in Table 1. For males, a significant association was found between exposure to violent games and friends’ aggression at T2. Thus, playing violent games was associated with more aggressive behavior of the friend 1 year later. There was also a significant correlation between aggression at T1 and friends’ aggression at T2, indicating that males’ own aggression at T1 related positively to friends’ aggression at T2. No statistically significant partner correlations were found for females.

Intraclass correlations describing similarity between partners on each study measure are presented on the diagonals in Table 1. Friends reported similar exposure to violent video games in male, but not female friendship dyads. Friends’ aggression scores at T1 and T2 were similar for both male and female dyads.

### 3.2 Primary analyses

**Figure 1:**
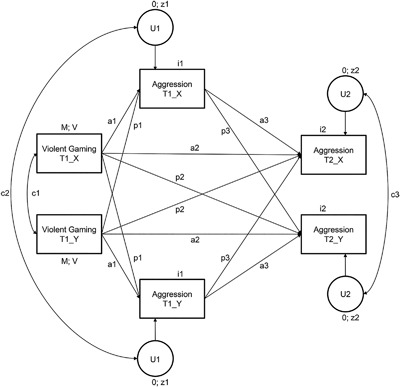
Actor‐Partner Interdependence Model predicting exposure to violent video games at Time 1 on aggression at Time 1 and Time 2 in indistinguishable friend dyads. All parameters are constrained to be equal for Adolescent X and Adolescent Y. a1, a2, a3 = actor effects; p1, p2, p3 = partner effects; c1 = Violent Gaming correlation; c2 = Aggression T1 residual correlation; c3 = Aggression T2 residual correlation. M = predictor means; V = predictor variances; i1, i2 = outcome intercepts; z1, z2 = residual variances; U1, U2 = outcome residuals. Violent Gaming T1_X = exposure to violent games of Adolescent X at T1, Aggression T1_X = aggressive behavior of Adolescent X at T1, Aggression T2_X = aggressive behavior of Adolescent X at T2. The same variables for Adolescent Y are indicated with extension _Y

Actor‐partner interdependence models (APIM; Kenny, Kashy & Cook, 2006) examined the effect of exposure to violent games at T1 on aggressive behavior at T2, while controlling for aggressive behavior at T1 for both dyad members (see Figure 1). To estimate the actor and partner effects for indistinguishable dyads within a structural equation modeling framework, equality constraints between dyad members were imposed on the variance and mean of the predictor variables, the actor effects, the partner effects, the intercepts, and the residual variances (Olsen & Kenny, 2006). The APIMs were performed with the “lavaan” package in R (Rosseel, 2012) using a robust maximum likelihood estimator (MLR).

First, gender differences were investigated using χ^2^ comparison tests. The model as shown in Figure 1 was run as a two‐group model with gender as the grouping variable. We compared the fit of an unconstrained APIM against a model where all three actor effects (a1, a2, and a3) and all three partner effects (p1, p2, p3) were constrained to be equal across gender. The unconstrained model showed good fit, χ^2^(24) = 25.97, *p* = .355, RMSEA = 0.04, CFI = 0.99, TLI = 0.98, while the model fit of the constrained model was mediocre, χ^2^(30) = 42.86, *p* = .060, RMSEA = 0.08, CFI = 0.91, TLI = 0.91. A χ^2^ difference test with Yuan‐Bentler correction showed the difference in model fit between the constrained and unconstrained model was significant, Δχ^2^(6) = 18.28, *p* < .01. Since forcing the parameters to be equal across gender significantly worsened the fit, the APIM was run separately for males and females.

The APIM for male dyads showed good fit, χ^2^(12) = 12.47, *p *= .409, RMSEA = 0.02, CFI = 0.99, TLI = 0.99. The standardized estimates for the actor and partner effects, as well as the correlations between residuals, are presented in Table 2. Results did not support an actor effect for exposure to violent games at T1 on aggression at T2. Thus, playing violent games did not positively predict changes in male adolescents’ own aggression one year later. There was, however, a significant partner effect of exposure to violent games at T1 on aggression at T2 for males. The partner effect indicated that male adolescents’ exposure to violent video games positively predicted changes in their best friends’ aggressive behavior 1 year later. In addition, there was a significant actor effect of aggression at T1 on aggression at T2, which shows that aggression was stable over time.

**Table 2:** Standardized estimates and correlations from APIM analysis for indistinguishable friend dyads separately for males and females

	Male dyads (*n* = 104)			Female dyads (*n* = 37)		
Variable	1	2	3	1	2	3
1. Exposure to violent games	.24*	.08	.06	.06	.07	.04
2. Aggression T1	.03	.50***	.56***	.12	.84	.56**
3. Aggression T2	.18*	−.04	.19	−.39	−.16	.21

Actor effects (a1, a2, a3) are above the diagonal, Partner effects (p1, p2, p3) are below the diagonal, and correlations (c1, c2, c3) are on the diagonal.

*p < .05, **p < .01, ***p < .001.

For female dyads, the fit of the APIM was adequate, χ^2^(12) = 13.37, *p* = .343, RMSEA = 0.06, CFI = 0.97, TLI = 0.97. Standardized estimates and correlations between residuals are presented in Table 2. No significant actor or partner effects were found for exposure to violent games, indicating that violent gaming was not linked to changes in females’ own, nor their friends’ aggression. There was a significant actor effect of aggression at T1 on aggression at T2, illustrating the stability of aggression over time.

**Figure 2:**
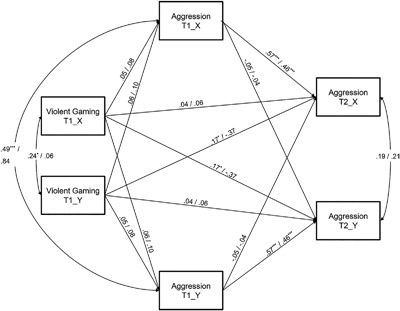
Actor‐Partner Interdependence Model predicting exposure to violent video games at Time 1 on aggression at Time 1 and Time 2 for indistinguishable friend dyads. The partner effect of violent gaming at T1 on aggression at T2 was allowed to vary across gender, all other actor and partner effects were set equal by gender. Standardized estimates and correlations given for male and female dyads, respectively. All parameters are constrained to be equal for Adolescent X and Adolescent Y. Violent Gaming T1_X = exposure to violent games of Adolescent X at T1, Aggression T1_X = aggressive behavior of Adolescent X at T1, Aggression T2_X = aggressive behavior of Adolescent X at T2. The same variables for Adolescent Y are indicated with extension _Y. *p < .05, *** *p* < .001

To determine the sources of gender moderation, the unconstrained two‐group model with gender as the grouping variable was compared to six separate APIMs where the actor and partner effects were constrained one by one. These six tests indicated that only the partner effect of exposure to violent games at T1 on aggression at T2 was moderated by gender. Compared to the fully unconstrained model, constraining this partner effect to be equal by gender significantly reduced model fit, Δχ^2^(1) = 13.16, *p* < .001, and the fit of the model was poor, χ^2^(25) = 40.72, *p* = .025, RMSEA = 0.09, CFI = 0.89, TLI = 0.87. A final model was run where the partner effect of exposure to violent games at T1 on aggression at T2 was allowed to vary across gender and all other actor and partner effects set equal by gender. The fit if this model was excellent, χ^2^(29) = 27.11, *p* = .566, RMSEA = 0.00, CFI = 1.00, TLI = 1.01. Standardized estimates and correlations between residuals are presented in Figure 2.

### 3.3 Moderation by gaming together or apart

Next, we investigated whether the partner effect was moderated by friends gaming together or apart. Given the small sample size of female dyads, this moderation was only tested in males. Means, standard deviations and correlations of the two groups are presented in Table 3. Independent sample *t*‐tests indicated that males in gaming‐together dyads scored significantly higher on exposure to violent games than those in gaming‐apart dyads, *t*(201) = 2.67, *p* = .008, *d* = 0.39. No significant mean‐level differences were found for aggression at T1, *t*(206) = 1.18, *p* = .239, *d* = 0.17, or aggression at T2, *t*(113.27) = 1.65, *p* = .103, *d* = 0.28. All correlations in Table 3 were compared by group using Fisher's *r*‐to‐*z* tests for independent correlations, but no significant differences were found between the correlation coefficients of gaming‐together and gaming‐apart dyads. This is a preliminary indication that the effects of violent gaming on aggression did not differ between groups.

**Table 3:** Spearman's ρ, means, and standard deviations for males in gaming‐together and gaming‐apart dyads

	Males gaming‐together (*n* = 132)			Males gaming‐apart (*n* = 76)		
Variable	1	2	3	1	2	3
1. Exposure to violent games	.29*	.08	.17	−.10	−.05	−.05
2. Aggression T1	−.02	.51***	.55***	.01	.54**	.50***
3. Aggression T2	.20*	.33***	.42**	.09	.18	.22
*M*	64.92	.37	.33	52.92	.23	.07
*SD*	31.22	1.08	1.05	29.33	1.03	.94

Intrapartner correlations are above the diagonal, cross‐partner correlations are below the diagonal, and intraclass correlations are on the diagonal.

*p < .05, **p < .01, ***p < .001.

To further investigate this, additional APIMs using a multiple group approach were conducted for the two groups of male dyads (gaming‐together and gaming‐apart). The model without any equality constraints between groups showed a good fit to the observed data, χ^2^(24) = 22.67, *p* = .539, RMSEA = 0.00, CFI = 1.00, TLI = 1.02. The fit of the APIM that constrained the partner effect between violent gaming and aggression at T2 to be equal between groups was also adequate, χ^2^(25) = 25.39, *p* = .441, RMSEA = 0.02, CFI = 0.99, TLI = 0.99. The difference between these models was not statistically significant, Δχ^2^(1) = 3.25, *p* = .071. Thus, contrary to our expectations, there was no evidence that the (partner) effect of violent gaming on friends’ aggression differed between dyads gaming together or apart.

### 3.4 Additional analyses

The sample used in the primary analyses was restricted to (a) individuals whose best friend nomination was reciprocated, and (b) individuals who played video games. Two sets of post‐hoc analyses were performed which relaxed each these inclusion criteria.

First, the sample size was increased by also including dyads in which peers nominated each other as second or third best friend. This increased the number of male dyads from 104 to 167 and the number of female dyads from 37 to 58. The APIMs, with identical specification as in Figure 1, indicated an excellent model fit for male dyads, χ^2^(12) = 10.68, *p *= .556, RMSEA = 0.00, CFI = 1.00, TLI = 1.01. Compared to the APIM restricted to reciprocal best friends, there were two important changes in the results. The partner effect of violent gaming on aggression at T2 was no longer significant, *β* = .08, *p* = .163 (see Table 4). Thus, adolescents’ exposure to violent video games did not predict friends’ aggressive behavior 1 year later. In addition, there was no longer a significant correlation between dyad members on exposure to violent games, *r* = .12, *p* = .167. For female dyads, the APIM including second or third ranked friend dyads also demonstrated excellent model fit, χ^2^(12) = 11.83, *p* = .460, RMSEA = 0.00, CFI = 1.00, TLI = 1.01. Compared to the APIM including only reciprocal best friends, the only change was that the stability effect of aggression from T1 to T2 was no longer significant, β = 0.29, *p* = .105. These additional analyses suggest a less robust pattern of statistically significant results when expanding the sample to include second and third ranked friends compared to the sample of best friends.

**Table 4:** Standardized estimates and correlations from APIM analysis for indistinguishable 1st, 2nd, or 3rd picked friend dyads separately for males and females

	Male dyads (*n* = 167)			Female dyads (*n* = 58)		
Variable	1	2	3	1	2	3
1. Exposure to violent games	.12	.02	.08	.26	−.00	.12
2. Aggression T1	.02	.44^***^	.54^***^	.09	.69	.29
3. Aggression T2	.08	.02	.15	−.29	−.02	.09

Actor effects (a1, a2, a3) are above the diagonal, Partner effects (p1, p2, p3) are below the diagonal, and correlations (c1, c2, c3) are on the diagonal.

^***^ *p *< .001.

Second, the sample size was increased by including adolescents who did not report playing video games at all. Non‐gaming participants received a score of 0 on exposure to violent games. However, from the original 496 male adolescents in the sample, no reciprocal best friend dyads in which both members did not play games could be identified. For females, adding reciprocal non‐gaming friends increased the number of total dyads from 37 to 87. Therefore, an APIM was conducted for female dyads in which the effect of exposure to violent games at T1 on aggressive behavior at T2 was estimated, controlling for aggressive behavior at T1. The fit for this model was excellent, χ2(12) = 10.53, *p* = .570, RMSEA = 0.00, CFI = 1.00, TLI = 1.04. There was a significant actor effect of T1 aggression on T2 aggression, β = 0.33, *p* < .05. There were significant correlations between dyad members for aggression at T1, *r* = .69, *p* < .05, and exposure to violent games *r* = .38, *p* < .01. However, the actor and partner effects of violent gaming on aggression remained nonsignificant. Thus, the inclusion of non‐gamers did not alter the results for male or female best friend dyads.

## 4 DISCUSSION

This longitudinal study examined whether the social context in which video games are played influences the association between exposure to violent games and aggressive behavior in middle adolescence. Dyadic analyses were conducted to investigate peer influence in best friend dyads. Exposure to violent games at T1 increased the aggressive behavior of best friends at T2 (*partner effect*) in male dyads, but not in female dyads. Thus, there was support for a peer influence effect of playing violent games on aggression for adolescent males.

Results did not indicate that the effect of violent gaming on friends’ aggression was moderated by dyads playing games either together or apart. Thus, there was no difference in partner effects between friends who played video games together and friends who played games separate from one another. We did not find that exposure to violent games increased adolescents’ own aggressive behavior 12 months later (*actor effect*), contrary to our expectations based on the General Aggression Model. This result seems in line with the meta‐analysis by Ferguson (2015), who argued that the deleterious influence of violent video games is minimal when looking at multivariate controlled effects. However, we did find a partner effect of violent gaming on aggression. These findings emphasize the importance of social influences from friends for the association between violent gaming and aggression.

Adolescent males’ exposure to violent video games predicted changes in aggressive behavior of their best friend 12 months later. We argue that this partner effect occurred because violent games increased deviancy training between friends. Research on deviancy training has associated peer interactions about antisocial behavior with an increase in antisocial behavior in adolescence (Dishion et al., 1997). Through social reinforcement of discussing and displaying positive affective behavior toward violent behavior in a video game, peers may promote each other's aggressive behavior. Regardless of adolescents’ own exposure to violent video games, friends’ exposure to violence in games could promote deviancy training, which in turn increases their own aggressive behavior.

The fact that a partner effect was found while a direct actor effect did not occur, raises interesting questions. We predicted an actor effect based on the GAM, which posits that repeated exposure to violent content creates aggression‐related knowledge structures in the long‐term (Anderson et al., 2010). One explanation for our findings is that the influence of peers is an essential factor for causing long‐term changes through violent gaming in this age group. Adolescents may be relatively resistant to influences of media content by itself, an idea supported by the fact that aggression is very stable over time (Breuer, Vogelgesang, Quandt, & Festl, 2015) and the relatively small effect size of violent media effects on behavior in longitudinal studies (Anderson et al., 2010; Greitemeyer & Mugge, 2014). At the same time, adolescents are particularly susceptible to peer influence effects (Brown & Larson, 2009; Steinberg & Monahan, 2007). Since the approval of peers is salient in adolescence, long‐term changes in aggression might occur only through moderation of the social context. We urge future research to replicate our findings, as well as test the influence of peers’ encouragement on the effects of playing violent video games using an experimental research design.

The lack of support for an actor effect of exposure to violent games on aggression is in contrast with two existing meta‐analyses (Anderson et al., 2010; Greitemeyer & Mugge, 2014). It is possible that our sample size was not large enough to detect a significant actor effect. Due to the focus on reciprocal best friend dyads as the unit of analysis, the number of participants in the current study was relatively low, particularly for females. Post‐hoc power analyses showed that around 86 female dyads would have been preferred in order to reach a power around 0.80. Thus, even considering the several adjustment that were made to increase sample size by including non‐gamers or 2nd and 3rd pick best friends, the null findings for females should be interpreted with caution.

Furthermore, the effect size has been reduced because we controlled for stability effects of aggression, removing a large portion of shared variance with the violent gaming predictor. The stability of aggressive behavior over time has been argued to reduce the influence of media effects on the long‐term (Breuer et al., 2015). In fact, when controlling for gender and initial level of aggression, the average longitudinal effect size from violent gaming on aggressive behavior dropped from *r *= .20 to *r* = .08 (Anderson et al., 2010), which is similar to the effect size found in this study. However, it is crucial to control for stability and eliminate variance due to selection effects in longitudinal studies of violent game effects. It speaks to the strength of the peer influence effect that violent gaming predicted friends’ aggression 1 year later, even after controlling for own and partner's aggression at T1.

Finally, we expected that the partner effect of exposure to violent games on aggressive behavior would be strongest in friend dyads who play video games together. This was based on the assumption that deviancy training would primarily take place between friends who regard gaming as a social activity. While all participants in the primary analyses played video games, friends who never play games together were assumed to talk less about their gaming experiences, reducing the amount of deviancy training. While results did not show a significant difference between males gaming together or apart, there were some indications that the partner effect was stronger in the gaming‐together dyads. Cross‐partner correlations between violent gaming and aggression at T2 were significant for gaming‐together dyads, but not gaming‐apart dyads, and there was a marginally significant change when comparing fit indices of the APIMs with constrained and unconstrained partner effect. Still, adolescent males showed increased aggression when their best friend played violent video games, regardless of whether they played together. We argue that exposure to violent games enhances deviancy training even in gaming‐apart dyads, as peers who do not play together can still discuss the violent content of video games they played. However, more research is needed to investigate the deviancy training mechanism. We particularly encourage observational research to test whether the number of game‐related antisocial interactions between peers is associated with increased aggression. Furthermore, instead of dichotomizing dyads as gaming together or apart, future research could also investigate a linear association between the amount of time spent playing violent games together and deviancy training.

In summary, these results show that the social context plays an important role in violent gaming effects. A peer influence effect was found even when controlling for stability effects of aggression and adolescents’ own violent gaming. We stress that the absence of an actor effect does not mean that individual effects of video game violence are negligible. Even if effects sizes are small, the prevalence of violent media makes it important to understand exactly when and how exposure influences youth. Several variables are known to moderate the association between exposure to violent video games and aggression, such as gender (Shibuya, Sakamoto, Ihori, & Yukawa, 2008), family environment (Fikkers, Piotrowski, Weeda, Vossen, & Valkenburg, 2013), or identifying with a media character (Krahé, 2014). This study emphasized that social context is an important addition to the list of potential moderators. Understanding these moderators is crucial for parents and policy makers alike in order to decide how to treat violent media for youth.

### 4.1 Strengths and limitations

Strong points of this study include the longitudinal design and the fact that the stability effect of aggression was controlled for. In addition, because aggression was measured with peer nominations instead of self‐report questionnaires, we removed possible single‐source measurement bias. The fact that these peer nominations were about real world behavior (pushing, shoving, and hitting others) adds to the external validity of the study as well.

There were several limitations. The sample consisted of reciprocal best friends, but the adolescents in these dyads may have differed from classmates who did not meet the inclusion criteria. For instance, the participants may have been better adjusted than their peers without a reciprocal friend in the classroom. This may have dampened the results, since deviancy training (Brechwald & Prinstein, 2011) and detrimental media effects (Krahé, 2014) have been shown to be stronger for at‐risk youth. Furthermore, post‐hoc analyses indicated that a peer influence effect of violent gaming on aggression for males was only present in reciprocal best friend dyads. When dyads with second and third ranked friends were included in the sample, the partner effect was not statistically significant. Thus, peer influence of violent gaming on aggression may only occur among close friends. Perhaps best friends spent more time together, allowing for more opportunities for deviancy training. In addition, similarity between friends’ violent gaming might play an important role as only best friends reported similar levels of exposure to violent games. The shared exposure to similar levels of violent content, together with ample opportunities to talk about them, may be a driving force behind deviancy training. Future research should investigate other social contexts in addition to best friends, such as siblings, parent‐child dyads, or peer groups. It will be particularly interesting to move beyond the dyadic level and investigate the impact of peer group norms on violent gaming and aggressive behavior using social network analyses.

There were relatively few female participants in the final sample, and the number of female dyads in which both members played games was small in particular. This is unsurprising, as surveys of nationally representative samples have shown that teen gamers are predominantly male (Lenhart et al., 2015) and males report more social motivations for picking up games (Lucas & Sherry, 2004; Olson, Kutner, & Warner, 2008). Yet the smaller sample calls for caution when interpreting results for females. Gender moderated the partner effect of exposure to violent gaming on aggression 1 year later, which justifies running analyses separate by gender. While a partner effect of violent gaming on aggression was not found for females, there is no theoretical reason to assume that females are immune from peer influence effects of violent media. Rather, gender differences may have occurred due to attributes of this specific study. For instance, aggressive characters in video games are often male (Smith et al., 2003) and boys might identify more easily with them, enhancing the learning effect (Konijn, Bijvank, & Bushman, 2007). In addition, we measured physically aggressive behavior, which is more prevalent for boys than girls (Lansford et al., 2012). Physical aggressive behavior was used since content analyses consider it to be the most prevalent form of aggression in video games (Smith et al., 2003) and it is likely the type of aggression most frequently modeled and rewarded (Anderson et al., 2010), as well as the most accessible target behavior for positive affect and encouragement from peers. Still, the unsupported actor effect of violent gaming on aggression and the absence of effects in female dyads may be due to our focus on physical aggression. Future research should examine peer influences of violent media on other forms of aggressive behavior, as well as aggressive cognitions, affect, and arousal. Furthermore, while the longitudinal approach is a strength of the study, it could also have created a bias in the data as we did not control for adolescents who stopped playing games throughout the year. Post‐hoc examination of the data indicated that there was little discontinuity of gaming behavior in males, but some females stopped gaming over the course of the study (1.9% and 29.8%, respectively). This presents an alternative explanation for the lack of support for the hypotheses in female dyads.

Another limitation was the way exposure to violent content in video games was measured. Participants indicated the genres they played, and each genre received a violence score that was multiplied by participants’ weekly playing time. Raters can reliably determine violent content per genre (Busching et al., 2015). However, we measured overall gaming frequency per week, which is less refined than the frequency per genre. Future studies should measure the frequency of each genre separately. In addition, we did not control for other possible effects of social context, such as the mode of play. A growing number of studies find that competitive gaming, compared to playing games alone or cooperatively, increases aggression. The effect of competition may influence aggressive behavior even more than the actual level of violent content in a game (Ewoldsen et al., 2012; Greitemeyer, Traut‐Mattausch, & Osswald, 2012) and playing competitive game genres has been associated with aggression, regardless of the amount of violent content in those genres (Adachi & Willoughby, 2016). Our measure of exposure to violent games might correlate with the amount of competitive play of adolescents, since violent game genres are often more competitive than non‐violent genres. Research that separates the effects of violent content and competitiveness in a game is an important next step. Furthermore, deviancy training might also enhance the influence of other forms of violent media and future research should look at the influence of positive affect of peers towards violence on television or in musical lyrics. Finally, it would be interesting to see if peers could also influence the association between exposure to prosocial content in games and prosocial behavior (Greitemeyer & Mugge, 2014).

## 5 CONCLUSION

This study showed that the social context influences the effect of violent video games on aggressive behavior. Adolescents’ exposure to violence in video games positively predicted the aggressive behavior of their best friend one year later. This (partner) effect was only found in male friendships, even when friends did not actually play video games with one another. We argue that violent games enhance deviancy training between peers, which increases their aggressive behavior. At the same time, no support was found for a direct (actor) effect of violent gaming on aggression. This is in contrast with several meta‐analyses that have demonstrated longitudinal effects of violent games on aggression, albeit small (Anderson et al., 2010; Ferguson, 2015; Greitemeyer & Mugge, 2014). The small sample size and controlled analysis might be responsible for the absence of a significant actor effect in the current study. Yet even hard to detect, small effects can still be of major practical significance when accumulated over time, and the prevalence of violent media urges us to better understand its impact on youths’ well‐being. We emphasize that peers play an important role in enhancing the effects of exposure to violent games, in particular for adolescents, and that the social context in which games are played is an important avenue for future research.

## CONFLICTS OF INTEREST

The authors declare that they have no conflict of interest.

## ENDNOTE

1 This is a conservative measure of gaming together, as one person's indication to not play games with their friend trumps the other dyad members’ indication. However, it was chosen because there were very few male friend dyads where both members indicated not playing games. There were only 8 dyads where both male friends indicated not playing games together, versus 96 dyads where at least 1 member stated to play games with his best friend.
